# Detection of atypical porcine pestivirus in Swedish piglets with congenital tremor type A-II

**DOI:** 10.1186/s12917-020-02445-w

**Published:** 2020-07-29

**Authors:** Hedvig Stenberg, Magdalena Jacobson, Maja Malmberg

**Affiliations:** 1 Section of Virology, Department of Biomedical Sciences and Veterinary Public Health, Swedish University of Agricultural Sciences, Box 7028, 75007 Uppsala, Sweden; 2 Department of Clinical Sciences, Swedish University of Agricultural Sciences, Box 7054, 75007 Uppsala, Sweden; 3 SLU Global Bioinformatics Centre, Department of Animal Breeding and Genetics, Swedish University of Agricultural Sciences, Box 7023, 75007 Uppsala, Sweden

**Keywords:** Congenital tremor, Type A-II, Atypical porcine pestivirus, Splay legs, Sweden, Swine, Piglets

## Abstract

**Background:**

Congenital tremor (CT) type A-II is a neurological disorder characterized by tremor of the head and body of newborn piglets. The suggested causative agent of the disease is the recently found atypical porcine pestivirus (APPV). The virus has been detected in piglets suffering from congenital tremor in central Europe, South and North America and in China but no studies has so far been performed in the Nordic countries. The overarching goal of this study was to investigate if APPV is present in the brain tissue of Swedish piglets suffering from congenital tremor.

From June 2017 – June 2018, 15 piglets from four Swedish farms with ongoing outbreaks of congenital tremor and 13 piglets with splay leg originating from four different farms, were investigated for presence of APPV RNA in brain tissue. Matched healthy control piglets (*n* = 8) were also investigated. Two APPV-specific RT-qPCR methods targeting the NS3 and NS5B region, respectively, were used. A retrospective study was performed on material from Swedish piglets with congenital tremor sampled in 2004 (*n* = 11) and 2011/2012 (*n* = 3) using the described APPV-specific RT-qPCR methods. The total number of piglets with signs of CT in this study was 29.

**Results:**

Atypical porcine pestivirus-RNA was detected in 93% (27/29) of the piglets suffering from congenital tremor. All piglets with congenital tremor from 2004 (*n* = 11) and 2012 (*n* = 3) were PCR-positive with respect to APPV, whereas, all of the healthy controls (n = 11) were negative. The piglets with congenital tremor sampled 2017–2018 had an odds ratio of 91.8 (95% CI 3.9128 to 2153.7842, z = 2.807, *P* = 0.0050) to test positive for APPV by qRT-PCR compared to the healthy piglets (Fishers exact test *p* < 0.0001). These findings make it interesting to continue investigating APPV in the Swedish pig-population.

**Conclusion:**

This is the first description of atypical porcine pestivirus in piglets suffering from congenital tremor type A-II in Sweden and the Nordic countries. The virus has been present in the Swedish pig population since at least 2004.

## Background

Congenital tremor (CT) is a neurological disorder that affects newborn piglets. The disease is characterized by tremors of the head and body, and may in severe cases be complicated by ataxia that exacerbates the piglets’ ability to move and suckle, resulting in reduced growth rate or increased pre-weaning mortality [1–3]. The neurological signs are caused by impaired saltatory transmission due to hypomyelination of the central nervous system (CNS) [3–5]. At present, congenital tremor is divided into six sub-types: type AI-AV and a B-type. By definition, all A-forms have hypomyelination of the CNS as the main histopathological finding, whereas the B-form presents with identical clinical signs but without histopathological lesions [1, 3–5].

For over half a century, congenital tremor type A-II was attributed to an unidentified virus [2], but in 2016 it was associated with the recently discovered atypical porcine pestivirus (APPV) [2, 6–8]. Atypical porcine pestivirus is a ssRNA+ virus that belongs to the species *Pestivirus K* in the genus *Pestivirus* within the family *Flaviviridae* [9, 10]. The genome of APPV is ~ 10.8–11.5 kb and up until now about 20 complete genomes have been published [11]. Interestingly, the genetic variability of the APPV isolates is very high also within the same geographic region [6, 12].

In two independent experiments with pregnant sows, APPV PCR-positive material was used to induce congenital tremor in piglets [7, 8]. Since the discovery of APPV in the US [9], the virus has been described in both diseased and healthy domestic pigs in Europe, Asia and in South and North America [6, 9, 13–18] and in serum as well as faecal samples from wild boars sampled in central Europe [14, 19, 20]. Atypical porcine pestivirus has also been detected in stored material from historical outbreaks of congenital tremor the oldest detection currently being from samples stored in 1997, originating from Spanish piglets suffering from congenital tremor [14].

Interestingly, in the experimental infections described above, splay leg was induced in 0–40% of the piglets born from the sows inoculated with APPV [7, 8]. This prevalence was an unusual observation of splay leg since the syndrome typically occurs only as sporadic cases [21], whereas congenital tremor commonly causes distinct, high-morbidity outbreaks [2, 15, 22].

Splay leg is characterized by impairment of the adducting muscles of the hindlimbs and, in severe cases, of the forelegs as well. This is attributed to hypomyelination of the spinal cord and the nerves innervating the affected muscles [23, 24]. The syndrome was first described in 1967 [23] and the hitherto proposed causal factors are numerous e.g., heritable gene defects, viral infections, various management factors, nutrition, and, fusarium toxin in the feed of the pregnant dam [21, 25–27].

There are three primary aims of this study: (1) to investigate the presence of APPV in brain tissue from Swedish piglets affected by congenital tremor or splay legs sampled 2017–2018; (2) to perform a retrospective study of the presence of APPV in historical material from Swedish piglets affected by congenital tremor; and (3) to perform a phylogenetic analysis of the obtained APPV sequences.

## Results

### Detection of APPV in piglets with congenital tremor

Atypical porcine pestivirus-RNA was detected by qRT-PCR in 93% (27/29) of the piglets suffering from congenital tremor. All piglets exhibiting signs of congenital tremor from 2004 (*n* = 11) and 2012 (*n* = 3) were PCR-positive with respect to APPV. Of the piglets sampled in 2017–2018, 87% (13/15) of the samples were positive with respect to APPV. Interestingly, the two piglets with congenital tremor that were negative with respect to APPV originated from the same farm (Farm F) sampled in 2018. All of the healthy controls sampled in 2017–2018 (*n* = 8) and the healthy controls sampled in 2012 (*n* = 3) were PCR-negative with respect to APPV.

The piglets with congenital tremor sampled 2017–2018 had an odds ratio of 91.8 (95% CI 3.9128 to 2153.7842, z = 2.807, *P* = 0.0050. MEDCALC®) to test positive in brain tissue for APPV by qRT-PCR as compared to the healthy piglets. Fishers exact test for the same sample gave a *p* < 0.0001 (Sergeant, ESG, 2019. Epitools epidemiological calculators. Ausvet Pty Ltd. Available at http://epitools.ausvet.com.au).

To get a clear overview of the Ct-values, all values were ranked in accordance with other publications; Ct-values (cycle quantification values) were graded as high (Ct < 28), moderate (Ct 28–33) and low (Ct 33–40) [14, 28]. Table 1 presents the mean Ct-values obtained from the piglets at each farm. The specific Ct-values obtained from each piglet are presented in the supplementary material in the file “cq_values_congenital_tremor_appv.docx”.

**Table 1:** A summary of the Ct values obtained from the piglets with congenital tremor from the six farms

Farm	Year	Location	Number of pigs	Ct-value
A	**2004**	Central Sweden	n = 11	low
B	**2012**	Central Sweden	n = 3	low – high
C	**2017**	Central Sweden	n = 3	high
D	**2017**	South of Sweden	n = 5	moderate – high
E	**2018**	Central Sweden	n = 5	high
F	**2018**	Central Sweden	n = 2	undetectable

**Fig. 1:**
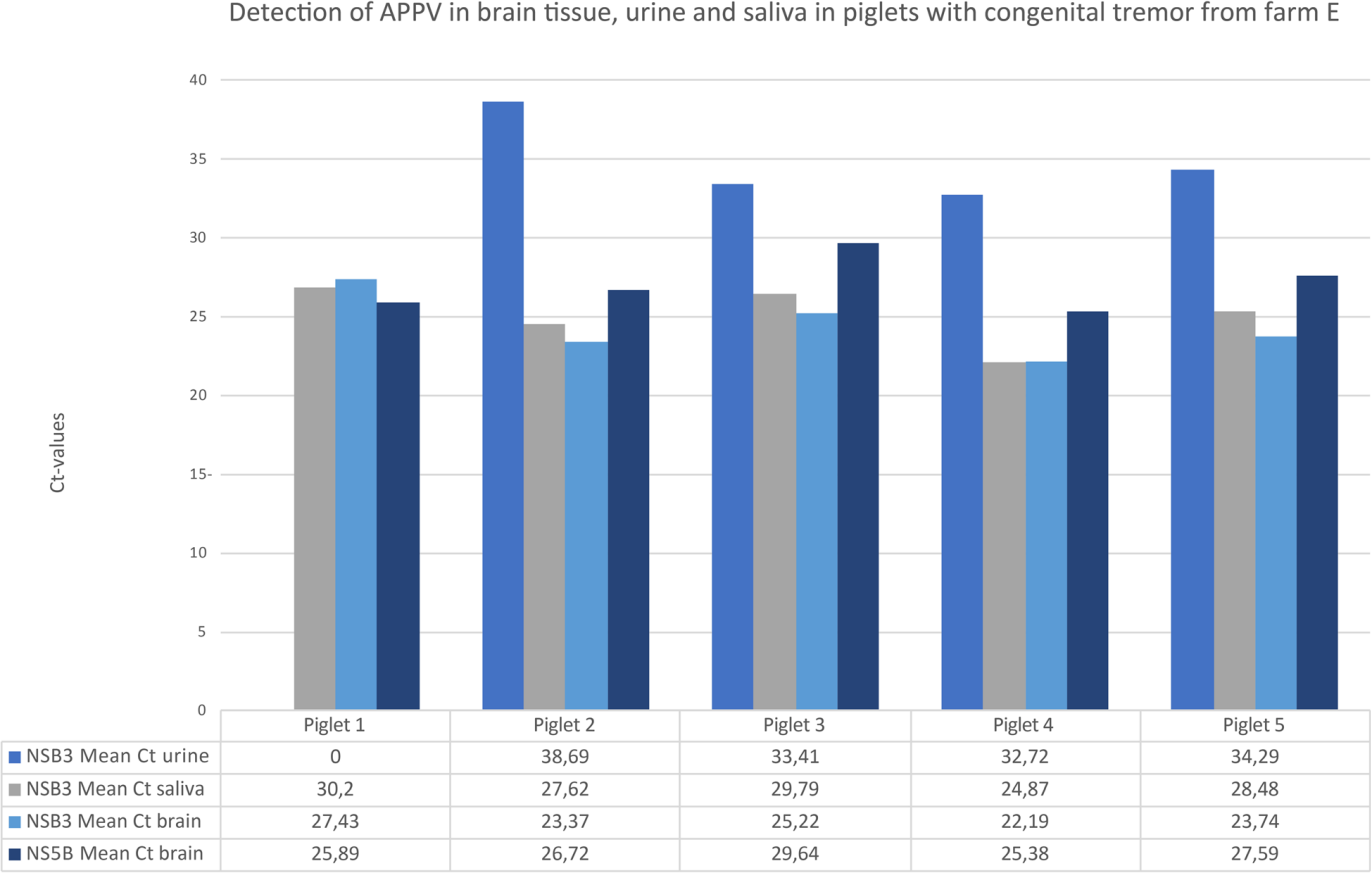
Five piglets with congenital tremor collected at farm E were screened for APPV-RNA in urine and saliva. RNA from atypical porcine pestivirus was detected with qRT-PCR in the saliva from 5/5 piglets and in the urine from 4/5 piglets. The Ct-values obtained from the brain-tissue are presented for comparison

As shown in Fig. 1, APPV-RNA was also detected by qRT-PCR in urine (in 4/5 piglets) and in saliva (in 5/5 piglets) from piglets with signs of congenital tremor.

### Sequence and phylogenetic analysis

The four samples from Farm B, C, D and E generated between 775 and 812 nucleotides from the APPV NS3 protein: Farm B (ID: LR700964, accession ERS3734195) 775 bp, Farm C (ID: LR700965, accession ERS3734196) 807 bp, Farm D (ID: LR700966, accession ERS3734197) 810 bp and Farm E (ID: LR700967, accession ERS3734198) 812 bp. All generated sequences have been deposited at the European Nucleotide Archive at EBI. The nucleotide identity among the four sequences ranged from 88.3 to 98.8%. The sequence Farm C (ID: LR700965) and Farm B (ID: LR700964) shared the highest identity at the nucleotide level at 98.8%.

Both Farm C (ID: LR700965) and Farm B (ID: LR700964) displayed the highest identity at the nucleotide level, 97.3%, to two sequences obtained from Chinese domestic pigs (GenBank accession MH378079.1 and MH509410.1). The other two Swedish sequences, Farm D (ID: LR700966) and Farm E (ID: LR700967), shared 97.4% identity at the nucleotide level. These two sequences, from Farm D and Farm E, both shared the highest nucleotide sequences identity, 96.2 and 95.6%, respectively, with a sequence obtained from a Spanish wild boar (GenBank accession LT855204.1).

**Fig. 2:**
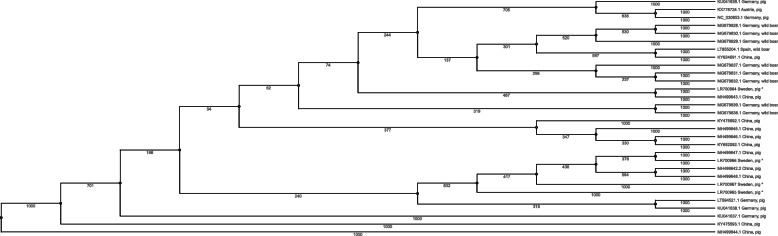
Phylogenetic tree of APPV-sequences, the Swedish sequences marked * cluster with sequences from domestic pigs from China as well as with wild boar from Germany

In the phylogenetic tree the Swedish sequences (marked * in Fig. 2) clustered with sequences from domestic pigs from China as well as with wild boar from Germany.

### Pathology

Of the 15 piglets sampled 2017–2018, all piglets with congenital tremor and the healthy controls had milk in their ventricles, whereas the ventricles of the piglets with splay leg were empty. No gross lesions were recorded at necropsy. Necropsies were also performed on all 11 piglets from 2011 but no gross lesions were recorded. These 11 piglets were also PCR-negative for PCV-2.

No necropsies were done on the 6 piglets sampled 2012, although the brains were subjected to histopathological investigation. In the piglets with clinical signs of CT, mild to moderate vacuolar changes of the white matter were observed in the cerebrum, brain stem, and cerebellum [29].

### No detection of APPV in piglets with splay leg

All of the piglets with splay leg (*n* = 13) sampled in 2017–2018 were PCR-negative in the brain tissue with respect to APPV.

## Discussion

This is the first description of APPV in piglets with congenital tremor type A-II in Sweden and in the Nordic countries. Atypical porcine pestivirus was detected by qRT-PCR in 27/29 samples of brain tissue obtained from 29 piglets with congenital tremor. The clinical samples were collected from five different farms between 2004 and 2018. Hence, this study provides evidence of APPV being present in Swedish pigs with congenital tremor type A-II since at least 2004. The virus was, however, not detected in the brain tissue of the healthy control piglets or the piglets with splay leg. Thus, in this study we found no evidence for APPV as the causative agent of splay leg in Swedish piglets.

One previous report has suggested astrovirus as a possible causative agent of congenital tremor type A-II in Swedish piglets but this report should be interpreted with caution since the virus was detected in both healthy and diseased animals [29]. However, the presence of APPV in the brain tissue of these astrovirus-positive piglets with congenital tremor and absence of APPV in healthy piglets provides evidence of astrovirus being an incidental finding, or being present as a co-infection, rather than the causative agent.

There are some proposed differential diagnoses to tremor in piglets e.g. PMWS, Aujeszky’s disease, porcine reproductive and respiratory syndrome (PRRS), aflatoxicosis, classical and African swine fever. Since Sweden has active surveillance programs and are deemed free from for Aujeszky’s disease, porcine reproductive and respiratory syndrome (PRRS) as well as classical and African swine fever, and PMWS (PCV-2) not are supposed to cause congenital tremor [30–33] we speculate that APPV is a causative agent of congenital tremor in Swedish piglets as well as in other countries [34–36].

The shedding of atypical porcine pestivirus in urine and saliva is in line with other publications [8, 15], but our study is the first report where a commercial viral swab was used. When more details of the shedding of APPV are identified, saliva sampling could in the future be an easy and cost-effective way to screen large groups of pigs for the virus.

The analysis of the sequences obtained from the APPV-positive piglets confirms the findings by others, that APPV is a genetically variable virus with no clear geographic clustering [37]. Interestingly, the Swedish sequences show nucleotide identity not only to sequences from domestic pigs in China but also to sequences from APPV in wild boars in Spain and Germany. Hence, a screening and phylogenetic analysis of APPV in the Swedish wild boar population would be highly interesting when trying to elucidate a possible route of transmission within Europe.

Since this is the first description of APPV in piglets with congenital tremor type A-II in Sweden, further studies are needed to determine the prevalence of APPV in the Swedish pig population. In addition, the occurrence as well as the mechanism of potential co-infections of other viruses and APPV should be investigated.

## Conclusion

This is the first description of atypical porcine pestivirus in piglets with congenital tremor type A-II in Sweden and the Nordic countries. The virus has been present in the Swedish pig population and been causing congenital tremor in piglets since at least 2004. Interestingly, the virus was not detected in piglets suffering from splay leg or in the healthy control piglets.

## Methods

### Clinical cases and sample collection

All animal studies were approved by the ethical committee of Uppsala 2017-02-10 (Dnr 5.8.10–00431/2017) and the owners of the herds gave informed consent prior to the start of the study.

**Fig. 3:**
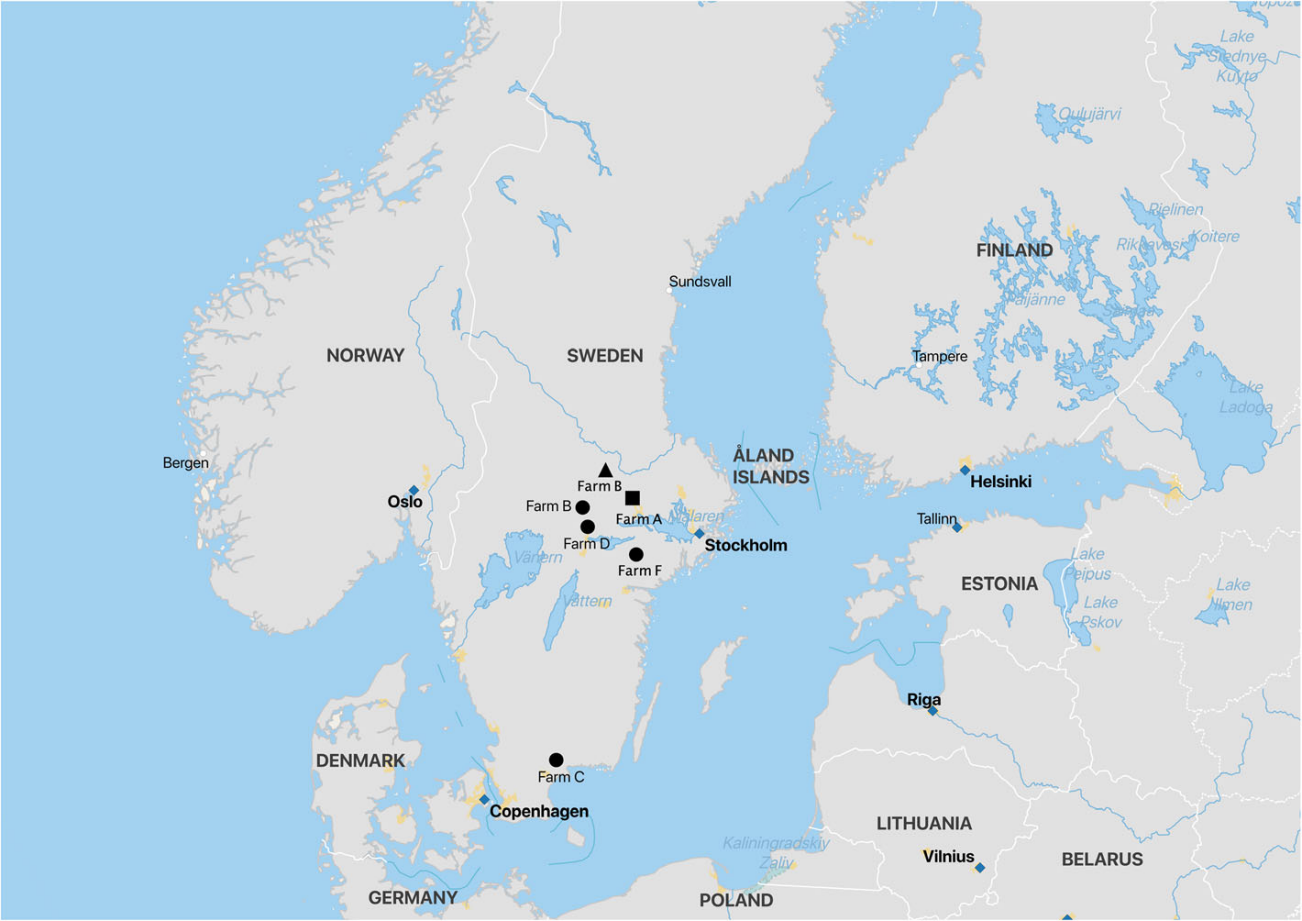
A map of Sweden showing the location of the farms were the piglets with congenital tremor were sampled. The map is made using QGIS (QGIS Geographic Information System. Open Source Geospatial Foundation Project. http://qgis.org)

During the period from June 2017 to June 2018, 15 piglets were obtained from four Swedish farms with ongoing outbreaks of congenital tremor. Of these piglets, 13 piglets were aged 1–2 days and two piglets were aged 5 days. All piglets were in good general condition with moderate to severe signs of congenital tremor. Three of the four farms were located in the central part of Sweden, with the remaining farm being located in the south of Sweden. The farms are marked on the map in Fig. 3. During the same period, 13 piglets aged 1–2 days old suffering from splay leg were obtained from four different farms located in the central part of Sweden. Most of these piglets had decreased demeanour. Piglets from the same farms and sows at their next farrowing were included as healthy controls; eight 1-day-old piglets in good condition were obtained. In cases where the original sow was unavailable, a piglet born to a sow from the same farrowing group was sampled. None of the sampled farms had any documented contact with each other and the outbreaks were separated in time, or had simultaneous outbreaks of congenital tremor and splay leg.

The piglets were transported to the pathology section at the Swedish University of Agricultural Sciences in Uppsala, Sweden. The piglets were sedated with an intramuscular injection of tiletamine and zolazepam (Zoletil®, Virbac, Carros, France) and a blood sample was obtained from the jugular vein. From the five piglets originating from farm E urine and saliva were also collected during sedation using commercial ESwabs (Copan Italia Via Perotti, Italy). All piglets were euthanized by an intraperitoneal injection of pentobarbital (Allfatal vet. Apotek Produktion & Laboratorier AB, Malmö, Sweden) with necropsy being performed within minutes.

Samples from the brain, spinal cord, saliva, urine, hearth, lung, quadriceps muscle, kidney, liver, spleen, ventricle, duodenum, jejunum, ileum, caecum, and colon were sampled and immediately put on dry ice. The tissue samples were then stored at − 80 °C. Corresponding tissue samples were fixed in 10% formaldehyde for future studies.

### Retrospective study

A retrospective study was carried out on material from piglets sampled in 2004 (*n* = 11) and 2011/2012 (*n* = 6). The samples from 2004 consisted of serum originating from eleven piglets affected by congenital tremor. The samples were collected from one litter of piglets originating from a farm located in the central part of Sweden. Necropsies were performed on all 11 piglets from 2004 with no records of gross lesions. These 11 piglets all tested negative for PCV-2.

The samples from 2012 consisted of brain tissue collected at the end of 2011 and beginning of 2012 from piglets on one farm during an ongoing outbreak of congenital tremor. Three newborn piglets with congenital tremor were euthanised and sampled at the farm. When the outbreak had ceased, three healthy newborn control animals from the same farm were euthanised similarly. The brains were collected and subjected to histopathological investigation, but no complete necropsies of the bodies were performed. Brain tissue from all six piglets tested PCR-positive with respect to porcine astrovirus [29]. In the piglets with clinical signs of congenital tremor, mild to moderate vacuolar changes of the white matter were observed in the cerebrum, brain stem, and cerebellum [29].

Both the serum and brain samples were stored at − 80 °C for future investigations.

### Sample preparation, RNA isolation, qRT-PCR (quantitative reverse transcription-PCR) and sequence analysis

The brain samples were cryolyzed using a Precellys tissue homogenizer (Bertin Corp. Rockville, MD, USA), RNA was extracted from all samples through a trizol-phenol-chloroform protocol, and cleaned using the GeneJET RNA kit (ThermoFisher Scientific, Waltham, MA, USA). In addition, RNA from the sera was extracted using the same protocol but without homogenization. The APPV genome was detected using an APPV-specific RT-qPCR protocol based on the QuantiTect Probe RT-PCR kit (Qiagen, Hilden, Germany) as described by [6] with a primer-pair targeting the NS3 encoding region of the APPV genome. The assay was run in duplicate under standard conditions on a Bio-Rad CFX96™ Real-time system in a C1000 Touch™ thermal cycler (Bio-Rad, Hercules, CA, USA) with a plasmid containing the NS3 encoding region of the APPV genome as a positive control. One primer and one probe, denoted “Swe” in Table 2, were slightly modified as compared to the protocol by [6] in accordance with [13], to better match the only described sequence of Porcine pestivirus in Sweden. All the samples were also analysed by an APPV-specific RT-qPCR targeting the non-structural protein NS5B in accordance with [12]. The RT-qPCR was run in duplicate under standard conditions using the qScript XLT One-Step RT-qPCR ToughMix (Quanta Biosciences, Gaithersburg, USA) on the above-mentioned Bio-Rad CFX96™ Real-time system in a C1000 Touch™ thermal cycler (Bio-Rad, Hercules, CA, USA).

**Table 2:** Primer and probe sequences used for qRT-PCR APPV detection, in accordance with previously published protocols, primers and probes [6, 12]. The primer and probe, denoted “Swe” are slightly modified in accordance with [13]

Oligo name	Sequence (5′-3′)
APPV-NS5B-303F	GTAGGGCGGATACAGAAATA
APPV-NS5B-385R	GGYACTTCCTCCATCATGG
APPV_5587-fw (NS3)	CAGAGRAAAGGKCGAGTGGG
APPV_5703_Swe-rev (NS3)	ACCATACTCTTGRGCCTGCAG
APPV_5087-fw (NS3)	GAAAGTGTCTGCCGCTTCATG
APPV-NS5B-336-FAM	AAATATTGGAAATYYATTGACAATTTGAC
APPV_Swe probe	ACTACTATCCTTCGGGGGTRGTRCCGA

### Sequence analysis

From each APPV-positive farm, the sample with the lowest Ct-value was selected for sequencing. A part of the NS3-gene was PCR-amplified using the primers APPV_5087-fw and APPV_5703_Swe-rev with the Invitrogen™ SuperScript™ IV One-Step RT-PCR-System, using the ezDNase™ Enzyme protocol (ThermoFisher Scientific, Waltham, MA, USA) according to the manufacturers’ instructions. The product was run on a 2% agarose gel stained with GelRed, visualized by UV-transillumination (GelDoc, Bio-Rad Laboratories, Inc., Richmond CA, US), purified using the Thermo Fisher Scientific GeneJET Gel Extraction Kit) and Sanger-sequenced at Macrogen Inc. Europe (Amsterdam, NL).

To get a clear and readily understood format of the tree, the phylogenetic analysis was performed on 26 full and partial genome sequences covering the APPV NS3 sequences extracted from the GenBank. The tree was constructed using the MAFFT alignment tool and the PHYLIP Neighbor-Joining method with a bootstrap value of 1000 using the UGENE software [38]. A bayesian tree were also made using the MR Bayes tool within the UGENE software [38]. To confirm the tree’s constitution and clustering, additional Neighbor-Joining trees were constructed, as well as Bayesian trees made with the MR Bayes tool within the UGENE software [38]. The bayesian trees and the Neighbor-Joining trees were consistent with each other.

## Supplementary information

**Additional file 1.**

## Data Availability

The specific QC-values are published in the supplementary material. The sequences analyzed during the current study are available at the European Nucleotide Archive at EBI under accession numbers LR700964- LR700967.

## Abbreviation

APPV: Atypical porcine pestivirus
