# Supplementary material for: Detection of atypical porcine pestivirus in Swedish piglets with congenital tremor type A-II
Source: BMC Vet Res. 2020 Jul 29;16:260. doi: 10.1186/s12917-020-02445-w (PMC7389371; doi:10.1186/s12917-020-02445-w)
Supplement: Supplementary file 1 — Additional file 1. [file 12917_2020_2445_MOESM1_ESM.docx]

| Clinical disease | Farm | Id | Sampled, year | Ct Value  mean NS3 | Ct value  mean NS5B |
| --- | --- | --- | --- | --- | --- |
| Congenital tremor | A | 9128R-A1 | 2004-09-01 | 37,87 | no data |
| Congenital tremor | A | 9128R-A2 | 2004-09-01 | 34,62 | no data |
| Congenital tremor | A | 9128R-A3 | 2004-09-01 | 33,94 | no data |
| Congenital tremor | A | 9128R-A4 | 2004-09-01 | 33,05 | no data |
| Congenital tremor | A | 9128R-A5 | 2004-09-01 | 23,63 | no data |
| Congenital tremor | A | 9128R-A6 | 2004-09-01 | 34,13 | no data |
| Congenital tremor | A | 9128R-A7 | 2004-09-01 | 35,11 | no data |
| Congenital tremor | A | 9128R-A8 | 2004-09-01 | 34,34 | no data |
| Congenital tremor | A | 9128R-A9 | 2004-09-01 | 33,76 | no data |
| Congenital tremor | A | 9128R-A10 | 2004-09-01 | 35,23 | no data |
| Congenital tremor | A | 9128R-A11 | 2004-09-01 | 34,73 | no data |
| Congenital tremor | B | 2012 - 1 | 2011 | 31,19 | no data |
| Congenital tremor | B | 2012 - 2 | 2011 | 27,58 | no data |
| Congenital tremor | B | 2012 - 3 | 2011 | 20,23 | no data |
| Congenital tremor | C | 91216-1 | 2017 | 25,75 | 28,22 |
| Congenital tremor | C | 91216-2 | 2017 | 26,04 | 26,85 |
| Congenital tremor | C | 4975 - 1 | 2017 | 21,29 | 21,04 |
| Congenital tremor | D | M1 | 2017 | 28,15 | 27,2 |
| Congenital tremor | D | M2 | 2017 | 28,02 | 26,78 |
| Congenital tremor | D | M3 | 2017 | 26,53 | 25,82 |
| Congenital tremor | D | M4 | 2017 | 23,22 | 22,26 |
| Congenital tremor | D | M5 | 2017 | 30,08 | 29,05 |
| Congenital tremor | E | 17034-1 | 2018 | 27,43 | 25,89 |
| Congenital tremor | E | 17034-2 | 2018 | 23,37 | 26,72 |
| Congenital tremor | E | 17034-3 | 2018 | 25,22 | 29,64 |
| Congenital tremor | E | 17034-4 | 2018 | 22,19 | 25,38 |
| Congenital tremor | E | 17034-5 | 2018 | 23,74 | 27,59 |
| Congenital tremor | F | 886716 | 2018 | 0 | 0 |
| Congenital tremor | F | 12017 | 2018 | 0 | 0 |
